# Progressive shingles in a toddler due to reactivation of Varicella Zoster vaccine virus four days after infection with SARS-CoV-2; a case report

**DOI:** 10.1186/s12879-023-08809-5

**Published:** 2023-12-06

**Authors:** Christine Miller, Emma Taylor-Salmon, Leonard Emuren, Marie Landry, Anne Gershon, George Miller

**Affiliations:** 1 Department of Pediatrics, Section of Infectious Diseases and Global Health, Yale University School of Medicine, 464 Congress Ave, New Haven, CT 06519 USA; 2 Department of Internal Medicine, Section of Infectious Diseases, Yale School of Medicine, New Haven, CT USA; 3 Department of Laboratory Medicine, Yale School of Medicine, New Haven, CT USA; 4 Department of Pediatrics, Vagelos College of Physicians and Surgeons, Columbia University, New York, NY USA

**Keywords:** COVID-19, Herpes Zoster (HZ), SARS-CoV-2, Shingles, Pediatric, Varicella Zoster Virus (VZV)

## Abstract

**Background:**

Herpes zoster (HZ) is the clinical syndrome associated with reactivation of latent varicella-zoster virus (VZV). Several factors have been implicated to promote VZV reactivation; these include immunosuppression, older age, mechanical trauma, physiologic stress, lymphopenia, and more recently, infection with severe acute respiratory syndrome coronavirus-2 (SARS- CoV-2). Recent reports suggest an increase in the number of HZ cases in the general population during the global COVID-19 pandemic. However, it is unknown what proportion of HZ during the pandemic is due to reactivation of wild-type or vaccine-strain VZV.

**Case:**

Here we report the first known case of HZ concomitant with SARS-CoV2 infection in a 20-month-old female who was treated with a single dose of dexamethasone, due to reactivation of the vaccine-type strain of VZV after presenting with a worsening vesicular rash.

**Conclusion:**

In this case, we were able to show vaccine-strain VZV reactivation in the context of a mild acute symptomatic COVID-19 infection in a toddler. Being able to recognize HZ quickly and effectively in a pediatric patient can help stave off the significant morbidity and mortality associated with disease process.

## Background

VZV, a neurotropic human alpha herpesvirus, is responsible for Varicella (Chickenpox) during primary infection and HZ (Shingles) during reactivated infection [1]. After initial infection, the virus lies dormant within the cells of dorsal root ganglia for months to years. Under various conditions the virus can be reactivated, resulting in the secondary form, HZ. VZV vaccine, a live attenuated virus of the Oka strain, is also capable of causing both mild primary infection and reactivation [2]. Reactivation of HZ from the Varicella vaccine has been previously described; however Shingles from vaccine virus is a rare event [3].

SARS-CoV-2 elicits a variety of immunologic disturbances [4]. Lymphopenia is a common finding in patients with acute COVID-19 infections [4]. Additionally, T-cell disturbances/dysfunction have frequently been described [4, 5]. One prominent abnormality is skewing of the CD4+ T cell response to the Th2 phenotype characterized by increased production of the cytokines IL-2, IL-4 and IL-6. Several previous studies have described a proposed link between VZV, particularly HZ, and acute COVID-19 illness [6–9]. A leading hypothesis is that the immunologic disturbances induced by SARS-CoV-2 allows for latent VZV to become reactivated.

Here we describe a toddler who developed severe progressive Herpes Zoster due to reactivation of vaccine strain (vOka) a few days after an acute COVID-19 infection. This case should help pediatricians to understand the pathogenesis of Shingles in young children without a history of previous chickenpox or exposure to chickenpox.

## Case

A 20-month old female with a past history of gastroesophageal reflux, laryngomalacia, bronchiolitis, reactive airway disease, and perinatal intraventricular hemorrhage presented to clinic with the chief complaint of a recurrent rash. She tested positive for SARS-CoV-2 in January 2021, then again 1 year later on January 11th, 2022. She was seen in the Pediatric Emergency Department on January 12 and 13 2022 due to wheezing; on the 12^th^ she received a single dose of dexamethasone (0.6 mg/kg) for reactive airway disease likely triggered by acute COVID.

**Fig. 1:**
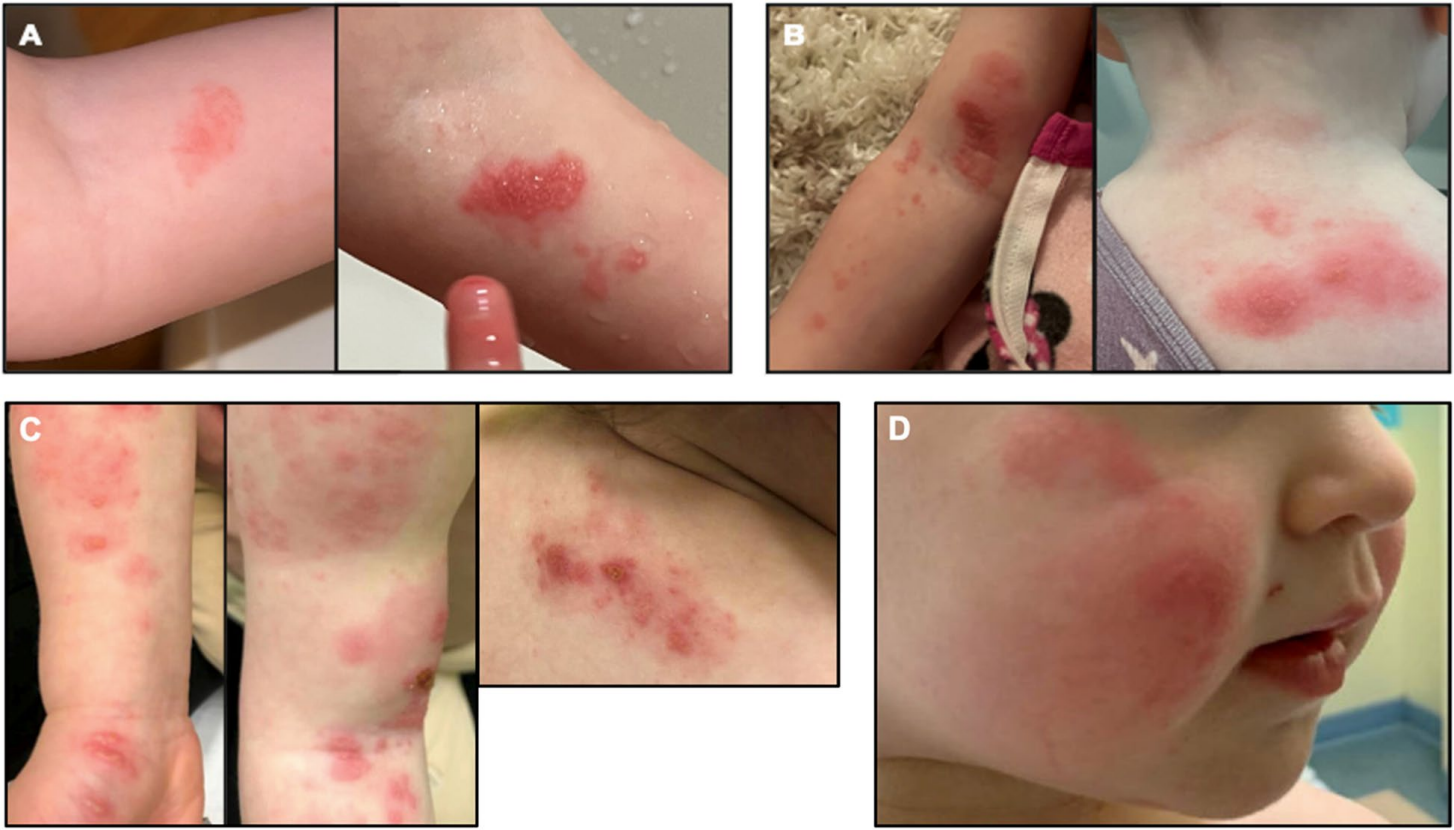
Progression of patient’s vesicular rash. **A** Initial presentation in antecubital fossa. **B** Progression of rash over the next several days, on to forearm and posterior neck. **C** Presentation to ED, now with lesions on thenar eminence and shoulder, with lesions in various stages of healing. **D** Readmission for facial rash

Four days after the patient tested positive for SARS CoV-2 (January 15th), she developed an erythematous, pruritic patch in her antecubital fossa; the rash subsequently developed vesicles that appeared fluid-filled (Fig. 1a). Two days later (January 17th), she was seen by her pediatrician, who prescribed topical acyclovir for possible HZ infection, as well as mupirocin due to concern for secondary bacterial infection. The following day (January 18th), the patient returned to her pediatrician due to concern that the rash was spreading to her back and forearm (Fig. 1b). At that time, the pediatrician switched her from topical to oral acyclovir, and sent a skin swab of the lesions for VZV and herpes simplex virus (HSV), and as well as superficial wound for bacterial culture.

Two days later (January 20, 2022), due to continued spreading of the rash and lack of improvement on oral acyclovir, the patient presented to the Pediatric Emergency Department. At this time, providers noted a vesicular rash on her right upper extremity and posterior neck, in various stages of healing that proceeded along a dermatomal distribution (Fig. 1c). The patient was prescribed an additional 5 days of PO acyclovir as well as cephalexin for superimposed impetigo.

The following day, the patient was seen in our Pediatric Infectious Disease clinic. At that time she was noted to have a vesicular and papular rash on her right arm, armpit, and upper back along the C5/6 dermatome that looked consistent with HZ. During this visit, the lesions no longer appeared to be exhibiting signs of a secondary infection, so cephalexin was stopped. Acyclovir was continued for an additional 5-days.

The patient returned to the ED 10 days after she was seen in our clinic, and 6 days after stopping oral acyclovir, due to erythematous rash on her right cheek (Fig. 1D). The lesions did not have any obvious vesicles at that time. She was admitted and started on IV acyclovir, with significant rapid improvement in symptoms. Repeat testing was negative for VZV but the decision was made to treat with valacyclovir for 5 days. Thereafter she did not develop any further lesions. In the interval of a year and a half after the episode, there were no recurrence of shingles or any other serious illness.

## Virology studies

The swab obtained in the pediatrician’s office was reported as positive for VZV by PCR, while HSV PCR was negative (Quest). A second specimen (obtained January 20^th^ while in the Pediatric Emergency Department) was sent to the Viral Diagnostic Laboratory at Yale-New Haven Hospital, was also positive for VZV DNA by PCR (probe sequence: (FAM)TCT CGA CTG GCT GGG ACT TGC G(TAMRA), forward sequence: 5′-TCT TGT CGA GGA GGC TTC TG-3′, reverse sequence: 5′-TGT GTG TCC ACC GGA TGA T-3’, [10]). This patient had no known exposure to VZV and was previously vaccinated with the VARIVAX vaccine on June 10th, 2021 in her right arm (Lot #T029534). Therefore, we investigated whether her lesions were caused by wild-type VZV due to infection from within the community, or by the Oka/Merck vaccine-strain, indicating a reactivation of latent virus from her prior vaccination. The VZV-positive swab of the lesions from the Emergency Department on January 20^th^ was sent to Merck for identification of the origin of the VZV strain as part of the US Varicella Zoster Identification Program (VZVIP). This test was carried out in Dr. Anne Gershon’s laboratory at Columbia University. The isolate was studied for five single nucleotide polymorphisms (SNPs), four of which are in ORF 62, which has mutations in positions 105705, 106262, 107252, and 108111 that distinguish wild-type Oka VZV from the Oka/Merck vaccine-strain, as well as a SNP marker at ORF38, a characteristic of clade 2 subtypes of VZV, from which the vaccine strain was derived. DNA from patient’s VZV was amplified by PCR and identified as the Oka/Merck vaccine strain by DNA sequencing.

## Discussion

To the best of our knowledge, this is the first known report of VZV vaccine-induced herpes zoster following recent symptomatic COVID-19 infection. Due to the temporal association of the HZ eruption and her COVID-19 infection, we questioned whether there may be an association.

This patient had been previously vaccinated with the VARIVAX vaccine, and reportedly had no known positive contacts with either Varicella or HZ, which raised our suspicion for vaccine reactivation. Importantly, during our investigation into the etiology of her VZV reactivation, we were able to sequence the strain and prove that it was indeed the Oka/Merck vaccine strain.

There have been previous reports linking COVID-19 infection to VZV reactivation [6–9, 11–17]. In a recent review by Diez-Domingo et al., 27 adult cases were described in which the majority of VZV reactivations occurred within 1 week of COVID-19 diagnosis (13/23 patients, 57%). This timing is similar to what was seen in our patient, with the eruption of the rash 4 days following a positive COVID-19 diagnosis. There have also been reports of severe, invasive manifestations of VZV following acute COVID-19, including meningitis in an 18-year-old otherwise healthy male [9] and encephalomyelitis in an 83-year-old male with chronic kidney disease [8]. Both of these published cases presented within 1 week of testing positive for mild symptomatic COVID-19 infections. In another review article [7], 29 patients, ranging in age from 7- to 86-years-old, were identified as having a temporal association of COVID-19 infection and HZ, with a large proportion presenting with HZ within 1 week of COVID-19 diagnosis. However, this is the first known case where the VZV causing reactivation was typed and shown to be due to a vaccine-strain of the virus.

Another recent study from a team in Brazil [6] found there to be an overall increase in the incidence in the number of HZ diagnoses from March to August of 2020, compared to March to August 2017 to 2019. Due to this increase in HZ cases during the early pandemic, the authors considered that there may be an association between HZ and COVID-19.

In terms of the pathophysiology, there has been speculation that lymphopenia and T-cell dysfunction resulting from COVID-19 infection may weaken the immune system in a way to allow for VZV to be reactivated [4, 5, 11, 12]. More specifically, a recent study investigating patients with Long COVID found there to be reactivation of certain herpes viruses [18]. Among other areas of immune dysregulation, Long COVID patients had a significant increase in VZV glycoprotein E antibody expression compared to healthy and convalescent controls which also corresponded to T cell activation and induction of IL-4 and IL-6 production [18]. Another proposed mechanism is the stress of the pandemic and COVID-19 infection can stimulate the neuroendocrine system, resulting in an imbalance of immune and inflammatory factors that could lead to VZV reactivation [12].

Although our patient did not have a complete blood count in any of her encounters for this illness, it is also possible that lymphopenia from the dexamethasone she initially received in the Emergency Department contributed to her development of VZV. There is evidence that even a single dose of dexamethasone can yield marked suppression of circulating lymphocytes [19]. Use of systemic steroids has also been linked to increased risk of VZV [20]. However there are no described cases of shingles due to vaccine-type virus following a single dose of dexamethasone.

In this case, we were able to show vaccine-strain VZV reactivation in the context of a mild acute symptomatic COVID-19 infection in a toddler. Being able to recognize the possibility of Varicella vaccine reactivation in the context of a recent COVID-19 infection is important to prevent delay in the subsequent diagnosis and treatment. VZV and its reactivation form, HZ, carry the potential of significant morbidity and mortality, especially within the young pediatric population. Prompt identification and treatment can stave avert worse disease sequelae. Reactivation of VZV is a rare occurrence within the pediatric population therefore we offer the above case to increase awareness.

## Data Availability

All data generated or analysed during this study are included in this published article.

## Abbreviations

HSV: Herpes Simplex Virus
HZ: Herpes Zoster
SARS-CoV-2: Severe Acute Respiratory Distress Syndrome Coronovirus-2
SNPs: Single Nucleotide Polymorphisms
VZV: Varicella Zoster Virus
VZVIP: Varicella Zoster Identification Program
